# Anterolateral Triangle: A Cadaveric Study with Neurosurgical Significance

**DOI:** 10.7759/cureus.2185

**Published:** 2018-02-12

**Authors:** Andre Granger, Ornella Bricoune, Tina Rajnauth, David Kimball, Heather Kimball, R. Shane Tubbs, Marios Loukas

**Affiliations:** 1 Department of Anatomical Sciences, St. George's University School of Medicine, Grenada, West Indies; 2 Icu/Anaesthetics, Eric Williams Medical Sciences Complex; 3 Neurosurgery, Seattle Science Foundation

**Keywords:** anterolateral triangle, far lateral, lateralmost, neurosurgery, dimensions

## Abstract

The anterolateral triangle is one of 10 surgical triangles of the cavernous sinus and serves as an important anatomic landmark for the skull base surgeon. There are few studies in the English literature that have precisely defined and measured the borders of the anterolateral triangle and little agreement has been made regarding the nomenclature within the English literature. A total of 12 midsagittally hemisected adult human cadaveric head halves were dissected to expose the anterolateral triangle. The triangle was defined and measurements of the anterior, posterior, and lateral borders were taken. The mean lengths and standard deviations of the anterior, posterior, and lateral borders were 8.3 ± 2.2 mm, 5.9 ± 2.0 mm, and 11.5 ± 2.9 mm, respectively. The mean area and standard deviation were 20.46 ± 9.30 mm^2^. The anterolateral triangle is helpful in understanding and planning surgical approaches to the cavernous sinus and middle cranial fossa. As such, normal anatomic relationships and the sizes of the anterolateral triangle must first be recognized to better access the pathologic changes within and around this region.

## Introduction

In general, there are 10 well-recognized surgical triangles surrounding the cavernous sinus. The borders of these triangles are roughly formed by cranial nerves (CN) II through VI and serve as important anatomic landmarks for the skull base surgeon. These triangles are helpful in understanding and planning surgical approaches to the cavernous sinus and middle cranial fossa [1]. The 10 triangles can be subdivided into three groups: cavernous sinus triangles, middle fossa triangles, and paraclival triangles. The cavernous sinus triangles consist of the (1) oculomotor, (2) clinoidal, (3) supratrochlear, and (4) infratrochlear (Parkinson’s) triangles. The middle fossa triangles consist of the (5) anteromedial, (6) anterolateral, (7) posteromedial (Kawase’s), and (8) posterolateral (Glasscock’s) triangles. The paraclival triangles consist of the (9) inferomedial and (10) inferolateral triangles [1].

**Figure 1:**
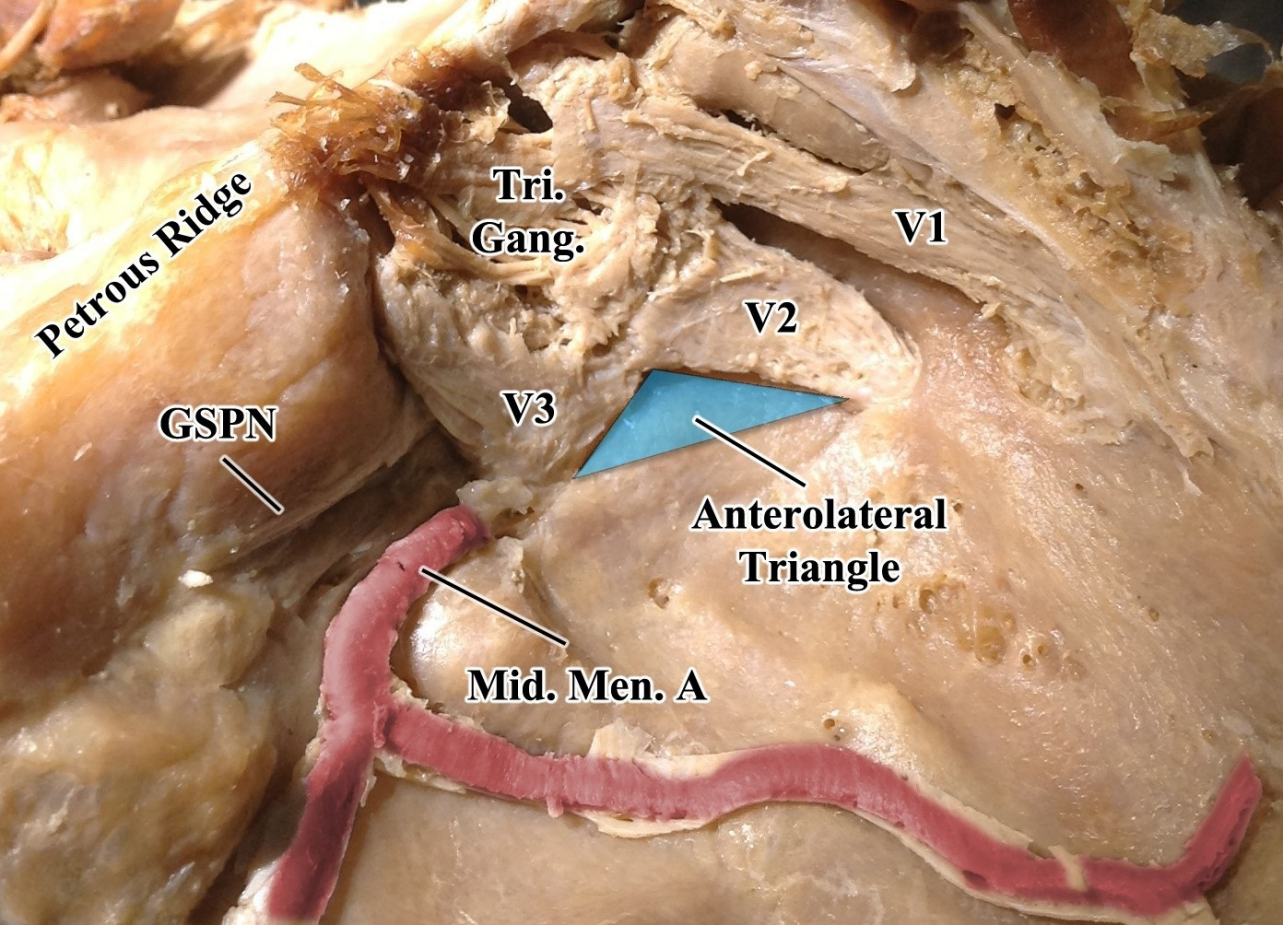
Superolateral view of the middle cranial fossa The dura mater has been removed to better visualize the underlying structures. The turquoise triangle represents the anterolateral cavernous sinus surgical triangle. The anterior border is defined along the posterior border of the maxillary division of the trigeminal nerve, from the intersection with the mandibular nerve to the posterolateral-most border of the foramen rotundum; the posterior border is defined along the anterior border of the mandibular division of the trigeminal nerve, from the intersection with the mandibular nerve to the anterolateral-most border of the foramen ovale; the lateral border is defined as a line connecting the posterolateral-most border of the foramen rotundum to the anterolateral-most border of the foramen ovale. GSPN, greater superficial petrosal nerve; Mid. Men. A., middle meningeal artery; Tri. Gang., trigeminal ganglion; V1, ophthalmic division of the trigeminal nerve; V2, maxillary division of the trigeminal nerve; V3, mandibular division of the trigeminal nerve.

The triangle is bound by the posterior border of the maxillary division of the trigeminal nerve, the anterior border of the mandibular division of the trigeminal nerve, and a line connecting the foramen rotundum to the foramen ovale (Figure 1). The triangle was originally described by Dolenc in 1989 as the lateral triangle and has since undergone several revisions in nomenclature [2-18]. Only a few studies have precisely defined and measured the borders of the anterolateral triangle [19-20], and little agreement exists regarding the triangle nomenclature and size. As a result, we aim to review and standardize the nomenclature, measure the borders, and provide a clear gross anatomic photographic depiction of the boundaries of the anterolateral triangle.

The anterolateral triangle contains the lateral wing of the sphenoid sinus, the vidian nerve, and the pterygoid region. Far anteroinferiorly, the maxillary sinus can be exposed, and posteriorly, the infratemporal Eustachian tube can be exposed under the lateral and medial pterygoid muscles. This far lateral triangle is the corridor toward the anterior infratemporal fossa approach for any of the infraorbital, maxillary, and pterygoid tumors, and the exposure of the epi- and parapharyngeal wall [21].

Komatsu et al. (2014) demonstrated that it was possible to endoscopically visualize the inferomedial temporal dura of the middle cranial fossa through the superior area of the anterolateral triangle. They thereby emphasized the consequential significance of this far lateral triangle for the preoperative planning of the surgical corridor in endoscopic endonasal approaches to the middle cranial fossa [22-23]. Likewise, Dolci et al. (2016) employed a transpterygoid endoscopic endonasal approach with two possible trajectories to the foramen ovale. The lateral to medial trajectory allows the feasible resection of benign tumors and a few malignant tumors [24]. Its route involves a violation of the lateral plate of the pterygoid process with the displacement of the lateral pterygoid muscle. For instance, tumors of the cavernous sinus that extend anterolaterally can be exposed through this triangular window [25]. Such tumors (e.g., adenomas) are candidates for endoscopic endonasal surgical removal because they are usually softer in consistency and, therefore, easier to suction out [23]. In the medial to lateral trajectory, the vidian nerve is located and tracked as it courses posteriorly near the mandibular division of the trigeminal nerve [24]. The vidian nerve can reliably be used as an anatomical landmark to the petrous internal carotid artery [26]. The internal carotid artery runs medial to the cranial nerves and posterior to the view of the triangle in the endoscopic endonasal view [23]. Specifically, the anterolateral triangle is used to gain access to critical structures, such as the anterior and lateral aspects of the C4 segment of the internal carotid artery, as well as the origin of the inferolateral trunk [27]. The sphenoid emissary foramen may also be visualized in this area. The medial wall of the anterolateral triangle can be opened to reach the lateral wall of the sphenoid sinus in certain cases [20]. For example, endoscopic endonasal closure has proven to be effective in the treatment of spontaneous meningoencephalocele of the lateral sphenoid sinus [28].

## Materials and methods

**Figure 2:**
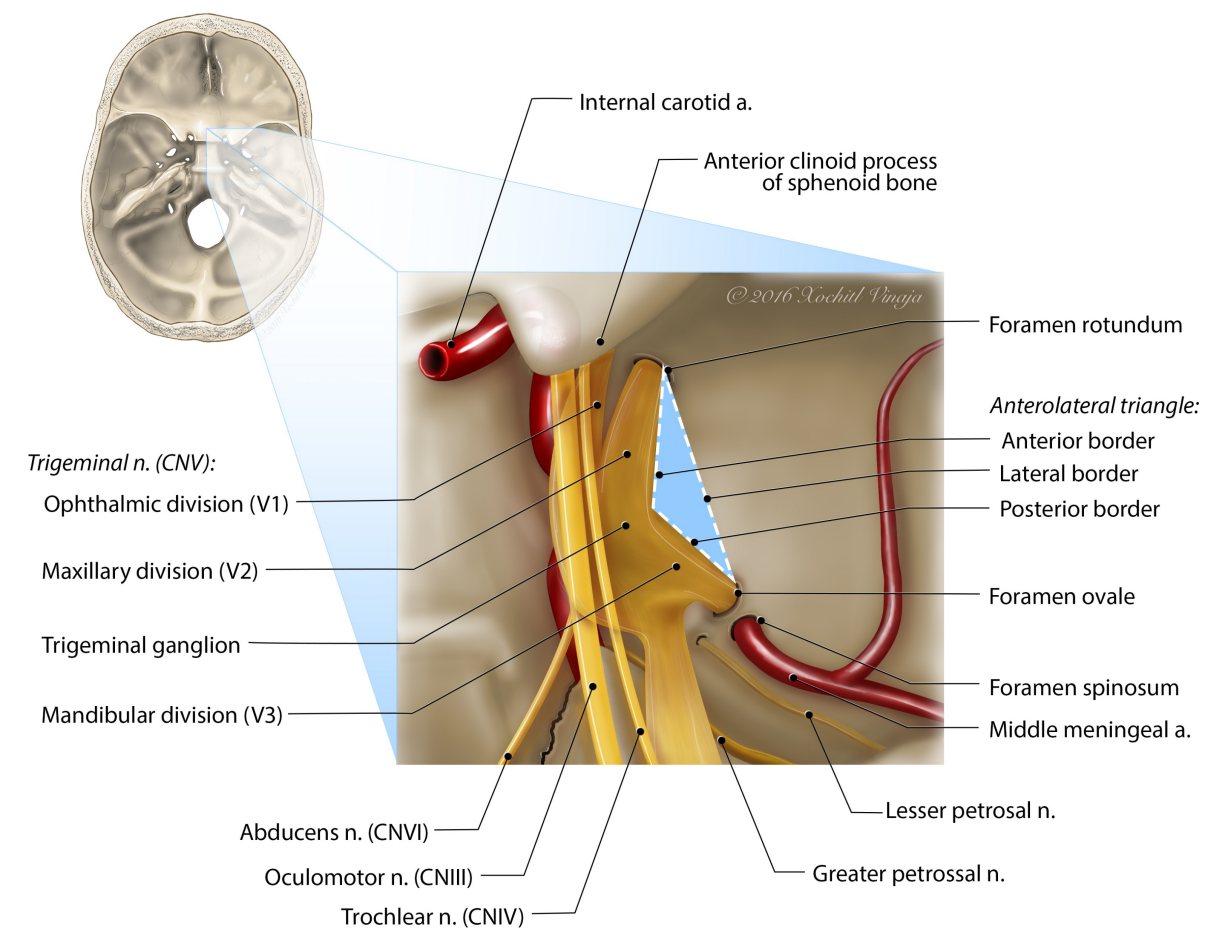
Illustration of the anterolateral triangle viewed superiorly This illustration highlights the borders and neighboring structures of the anterolateral triangle. The inset image is that of a skull base and can be used for orientation.

A total of 12 human adult formalin-fixed midsagittally hemisected cadaveric head halves were subjected to dissection at St. George’s University School of Medicine. The brains were previously dissected out in all specimens, leaving the cranial nerve roots, dura mater, and tentorium intact. Using a Seiler Evolution xR6 surgical microscope (Seiler Instruments, Missouri, US) and working laterally to medially, careful dissection was done to reflect away the dura mater overlying the floor of the middle cranial fossa, exposing the underlying structures. Particular care was taken to preserve the trigeminal nerve and its three divisions: the middle meningeal artery and the greater and lesser petrosal nerves. The anterolateral triangle was then identified and the borders defined. The anterior border was defined along the posterior border of the maxillary division of the trigeminal nerve, from the intersection with the mandibular nerve to the posterolateral-most border of the foramen rotundum. The posterior border was defined along the anterior border of the mandibular division of the trigeminal nerve, from the intersection with the maxillary nerve to the anterolateral-most border of the foramen ovale. The lateral border was defined as a line connecting the posterolateral-most border of the foramen rotundum to the anterolateral-most border of the foramen ovale (Figure 2). Measurements were taken of each of the borders using a dial caliper with a 1/10-millimeter precision.

A Google Books search was also done to identify different nomenclature used for what we described as the anterolateral triangle throughout various texts. We ran seven searches within Google Books, each with a different key term. The following search terms were used: “anteromedial triangle,” “anterolateral triangle,” mullan triangle (no quotes), “lateral triangle” cavernous sinus (no quotes), Dolenc triangle (no quotes), and “lateralmost triangle.” All results and gathered reference information for each relevant search result was reviewed to identify which related to the anterolateral triangle. The key terms were reviewed within the Google Books text of each search result and the corresponding triangle nomenclature was recorded.

## Results

The lengths of the anterior border (along the maxillary nerve) ranged from 5.1 mm to 11.5 mm, with a mean length and standard deviation of 8.3 mm and 2.2 mm, respectively. The mean length and standard deviation of the posterior border were 5.9 mm and 2.0 mm, respectively, and ranged from 3.3 mm to 8.8 mm. The lateral border length ranged from 7.1 mm to 16.5 mm, with a mean length and standard deviation of 11.5 mm and 2.9 mm, respectively. The area within the anterolateral triangle ranged from 5.31 mm^2^ to 38.53 mm^2^, with a mean area and standard deviation of 20.46 mm^2^ and 9.30 mm^2^, respectively (Table 1).

**Table 1:** Measurements of the anterolateral triangle Table showing the measurements obtained from the dissection of the anterolateral triangle 1 Left side 2 Right side 3 Measured along the posterior border of the maxillary division of the trigeminal nerve, from the intersection with the mandibular nerve to the posterolateral-most border of the foramen rotundum 4 Measured along the anterior border of the mandibular division of the trigeminal nerve, from the intersection with the maxillary nerve to the anterolateral-most border of the foramen ovale. 5 Measured along a line connecting the posterolateral-most border of the foramen rotundum to the anterolateral-most border of the foramen ovale. 6 Standard deviation

	Border Length (mm)			Area (mm^2^)
Specimen	Anterior^3^	Posterior^4^	Lateral^5^	
No. 1^1^	10.4	3.7	11.8	18.74
No. 2^1^	8.1	4.6	10.6	17.50
No. 3^2^	8.9	7.2	14.2	26.74
No. 4^1^	9.0	3.3	9.8	14.81
No. 5^2^	10.2	7.8	14.3	38.53
No. 6^1^	5.3	4.1	9.1	5.31
No. 7^1^	5.5	6.0	7.1	16.02
No. 8^1^	11.5	3.3	11.9	18.97
No. 9^1^	7.7	7.0	11.9	25.63
No. 10^2^	10.4	7.9	16.5	32.27
No. 11^2^	7.0	7.0	7.2	21.61
No. 12^1^	5.1	8.8	13.6	9.40
Mean ± SD^6^	8.3 ± 2.2	5.9 ± 2.0	11.5 ± 2.9	20.46 ± 9.30

Seventeen relevant text results with mention of the surgical triangle lying between V2 and V3, each using particular respective nomenclature in referring to the triangle, were found in our Google Books search (Table 2). Dolenc (1989) was the first to mention the triangle and referred to it as the “lateral triangle” [24]. Including Dolenc (1989), five (29%) of the 17 text results used the name “lateral triangle;” six (35%) texts used the name “far lateral triangle;” five (29%) texts refer to the triangle as the “anterolateral triangle;” one (6%) text refers to the triangle as the “lateralmost triangle.” Of note, the majority of the most recently published texts referred to the triangle as the “anterolateral triangle.”

**Table 2:** Nomenclature assigned to the anterolateral triangle Table showing the various terminologies used to refer to the anterolateral triangle 1 Search results from Google Books 2 Corresponding figure lists the "far lateral triangle"

Text^1^	Triangle Nomenclature
Dolenc, 1989 [2]	Lateral
Koos et al., 1993 [3]	Lateral
Day and Tschabitscher, 1996 [4]	Far Lateral
Loftus, 1996 [5]	Far Lateral
Eisenberg and Al-Mefty, 2000 [6]	Far Lateral
Robertson et al., 2000 [7]	Lateral
Fossett and Caputy, 2002 [8]	Lateral
Kobayashi, 2005 [9]	Anterolateral
Badie, 2007 [10]	Lateral
Pickard, 2008 [11]	Far Lateral
Dolenc and Rogers, 2009 [12]	Anterolateral
Sindou, 2009 [13]	Far Lateral
Wanibuchi et al., 2009 [14]	Far Lateral
Abdulrauf, 2010 [15]	Anterolateral
Banerji and Pauranik, 2010 [16]	Anterolateral^2^
Laws and Sheehan, 2011 [17]	Anterolateral
Quinones-Hinojosa, 2012 [18]	Lateralmost

## Discussion

Despite the first description of this triangle by Dolenc (1989) using the nomenclature “lateral triangle,” there was a shift in the literature regarding a more standardized and simplified nomenclature of the surgical triangles of the cavernous sinus. In particular, Dr. Al Rhoton, who is a leading expert in cranial microsurgical anatomy, uses the nomenclature anterolateral triangle when referring to the surgical space between V2 and V3 [1]. Four of our five most recently published texts in our Google Books search results referred to the triangle as the “anterolateral triangle.” It was, therefore, our decision to use the nomenclature “anterolateral triangle” when describing the space between the maxillary (V2) and mandibular (V3) division of the trigeminal nerve.

Two studies in the literature defined and measured the anterolateral triangle [19-20]. Watanabe et al. (2003) use the nomenclature “lateral triangle” when referring to this surgical space and report triangle border lengths from 12 Japanese adult cadaveric head-sides. The mean length of the anterior border was reported as 13.9 (± 3.1 mm), somewhat longer than our 8.3 (± 2.2 mm). The posterior boundary reported as 7.6 (± 2.9 mm) was also larger than our measurement 5.9 (± 2.0 mm). The lateral and final border, with a mean length of 15.5 (± 2.3 mm), also yielded shorter distances in our study measuring 11.5 (± 2.9 mm). Watanabe et al. (2003) reported the area and SD as 49.8 ± 15.5 mm^2^, almost double the size of our result (20.46 ± 9.30 mm^2^). In a similar study by Isolan et al. (2007), 18 cavernous sinuses were dissected to expose and measure the 10 surgical triangles within the area. Isolan et al. (2007) defined the triangle as the “anterolateral triangle.” The average measurements and SD of the anterior, posterior, and lateral borders of the triangle were 12.25 ± 0.67 mm, 11.59 ± 1.61 mm, and 10.80 ± 1.12 mm, respectively. The average area and SD of the triangle was 51.52 ± 4.25. As opposed to using calipers for measuring the area of the anterolateral triangle, Dolci et al. (2016) used stereotaxy and predetermined coordinates to calculate the area of the anterolateral triangle. Their study yielded an average area of 47.27 ± 5.27 mm^2^.

The aforementioned studies had measurements that were larger than our measurements. There may be several reasons for such observations. One such reason, however, inferred from the figures within both studies, may be due to different degrees of dissection between V2 and V3. That is, different amounts of dura mater removed from V2 and V3 may influence the perceived lengths of V2 and V3. In this study, every effort was made to dissect only dura and leave intact cranial nerves to obtain clean measurements (Figure 1).

## Conclusions

As one of the 10 well-recognized triangles surrounding the cavernous sinus, the anterolateral triangle, as it is most commonly called, serves as a critical window in endoscopic endonasal surgeries and for gaining access to other middle fossa structures. The mean lengths of the anterior, posterior, and lateral borders were 8.3 ± 2.2 mm, 5.9 ± 2.0 mm, and 11.5 ± 2.9 mm, respectively. These anatomical findings may aid in optimizing preoperative planning.
